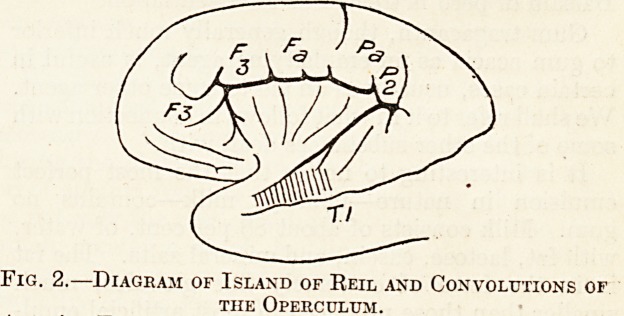# The Cortical Centre for Hearing

**Published:** 1908-02-22

**Authors:** Purves Stewart


### Notes on Current Neurology.

### THE CORTICAL CENTRE FOR HEARING.

By PURVES STEWART, M.D., F.R.C.P.

For a number of years clinical and pathological observations have generally been held to support the view that the cortical auditory centre is situated on the convex surface of the brain, in the upper temporal convolution (see fig. 1). In certain cases of congenital deafness this convolution has been stated to be atrophied, whilst destructive lesions of the convolution have been associated with loss of hearing, usually transient, in the opposite ear, and also, if the cortical lesion be left-sided, with word-deafness. On the other hand, cases of disease in the upper temporal gyrus have now and then been observed in which neither deafness nor word-deafness has occurred, and the explanation of these apparent exceptions has hitherto been obscure. Recently, however, Flechsig has thrown considerable light on the subject and has brought strong evidence to show that though the auditory centre is undoubtedly in the temporal lobe, its localisation in the upper temporal gyrus is only partly accurate. The main auditory centre, according to Flechsig, is in a part of the temporal lobe which is invisible when the brain is looked at in the ordinary way. The true cortical centre for hearing is in the anterior transverse temporal gyrus (sometimes called Heschl's convolution) on the upper or Sylvian surface of the temporal lobe, forming part of the operculum, continuous with the cortex of the Island of Reil. Heschl's convolution can only be seen by separating the walls of the Sylvian fissure (see fig. 2). It is continuous with the upper temporal gyrus, which was formerly considered as the main auditory centre.

Fig. 1.—Diagram of Convex Surface of Cerebrum. Vertical shading indicates localisation of Auditory Centre, as given in most text-books.

Flechsig bases his statements partly upon the results of the study of myelinisation of fibres in human embryos of various ages, and partly on the secondary degenerations which follow destructive lesions of Heschl's convolution. The projection fibres leading from the anterior transverse temporal gyrus into the sub-lenticular region of the internal capsule form a distinctive bundle, which may be regarded as the "auditory radiation," analogous to the "visual radiation" in the occipital lobe. The fibres of this auditory radiation are connected mainly with Heschl's convolution and only extend to a slight extent on to the convex surface of the brain towards the first temporal gyrus. If Flechsig's views are correct, and there seems no sound reason for rejecting them, we must regard Heschl's convolution as the true auditory convolution. It is interesting to note that in right-handed people this gyrus is larger and better developed on the left side of the brain than on the right; in fact, as a rule on the left side there is only one definite gyrus, the anterior, whilst on the right side there are both an anterior and a posterior transverse gyrus.

Fig. 2.—Diagram of Island of Reil and Convolutions of the Operculum.
Anterior Transverse Gyrus of Heschl is shaded vertically.

## Figures and Tables

**Fig. 1. f1:**
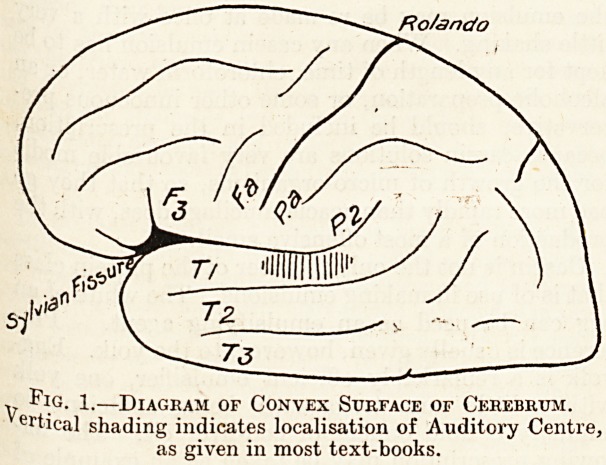


**Fig. 2. f2:**